# Hemodynamic characteristics and the occurrence of renal biopsy-related arteriovenous fistulas in native kidneys

**DOI:** 10.1007/s11255-016-1411-z

**Published:** 2016-08-31

**Authors:** Arkadiusz Lubas, Anna Wojtecka, Jerzy Smoszna, Piotr Koziński, Emilia Frankowska, Stanisław Niemczyk

**Affiliations:** 1 Department of Internal Diseases, Nephrology and Dialysis, Military Institute of Medicine, Warsaw, Poland; 2 Department of Radiology, Military Institute of Medicine, Warsaw, Poland

**Keywords:** Renal biopsy, Arteriovenous fistula, Resistance index

## Abstract

**Purpose:**

Renal biopsy-related arteriovenous fistula (RB-AVF), although usually asymptomatic, may sometimes result in serious clinical implications. The aim of the study was to prospectively evaluate the incidence of RB-AVF in native kidneys, together with the assessment of hemodynamic characteristics and the impact of the histopathological results of biopsy.

**Methods:**

The study included 138 patients (age 46.2 ± 15.2; 70 F, 68 M), who underwent percutaneous renal biopsy (PRB) of the native kidney. In all patients, 2D and color Doppler ultrasound was performed 24 h after PRB in order to exclude RB-AVF.

**Results:**

Bleeding complications in the form of hematomas were found in 136 patients (98.55 %), and 23 cases of RB-AVF were observed (16.67 %). RB-AVF group had an increased maximal hematoma diameter and reduced number of glomeruli in PRB. The segmental arteries supplying the fistulas are characterized by higher maximum flow velocity (FV) and a lower resistance index (RI) compared to the normal segmental arteries (difference 45.9 ± 20.0 cm/s and 0.252 ± 0.104, respectively). In the ROC analysis, RI ≤ 0.524 allowed to detect RB-AVF with a sensitivity of 91 % and specificity of 100 % (AUC 0.998, *p* < 0.001). In approximately 39 % of RB-AVF cases, 2D ultrasound detected a hyperechogenic ischemic area between the fistula and the renal capsule.

**Conclusions:**

Arteriovenous fistula is a quite frequent complication of native renal biopsy and can cause ischemia in the renal parenchyma detected by ultrasound. The arteries supplying the fistula are characterized by an increased flow velocity and reduced resistance index.

## Introduction

Percutaneous renal biopsy (PRB) is an essential tool in the diagnosis of diseases of the native and transplanted kidneys. The progress made in recent years, both in terms of biopsy devices, ultrasound techniques and equipment and the possibility of endovascular treatment, significantly improved safety of this procedure [1, 2]. Due to the technique and the ultrasound equipment necessary to monitor the procedure, in some centers, instead of nephrologists, PRB is more and more often performed by radiologists [3]. Clinical monitoring of a patient after PRB without radiological imaging as well as radiological approach without clinical data results in a significant discrepancy in the reported complications. In view of the fact that nearly 98 % of PRB complications occur within 24 h after the procedure and only 46 % after 4 h and 79 % after 8 h, it is recommended to monitor the patient until the next day [4]. One of the complications of PRB is an abnormal shunting of blood from the artery to the vena in the vicinity of the biopsy channel, namely post-biopsy arteriovenous fistula (RB-AVF). Generally, a majority of observed fistulas are clinically asymptomatic and close spontaneously. This fact, together with ignoring the diagnostic ultrasound or discontinuation of the use of the color Doppler (CD) imaging in clinically silent cases, contributes to reducing the incidence of RB-AVF. On the other hand, some sources report significant clinical complications in as many as 20–41 % of RB-AVF cases and the necessity of invasive treatment [5].

The aim of the study was to prospectively evaluate the incidence of RB-AVF in native kidneys, together with the assessment of hemodynamic characteristics and the impact of the histopathological results of biopsy.

## Methods

From 172 consecutive patients who underwent percutaneous renal biopsy of native kidneys in the period from January 2012 to March 2016 (51 months) in the Department of Nephrology, 138 Caucasian patients (age 46.2 ± 15.2; 70 F, 68 M) were qualified to the prospective observational study. The involved patients were monitored after PRB by ultrasound for the occurrence of iatrogenic arteriovenous fistula associated with PRB. The study was conducted in accordance with the Declaration of Helsinki. All participants enrolled in the study gave informed consent.

PRB was performed in patients previously qualified by an experienced nephrologist, with correct results of activated partial thromboplastin time (aPTT), international normalized ratio (INR), platelet count >100000/ml and blood pressure below RR 140/90 mmHg, without focal lesions in the lower pole of the left kidney in ultrasound examination. If used, all antiplatelets and anticoagulants were discontinued 7 days before the biopsy, except for low molecular weight heparin, administered not later than 24 h before the PRB.

Biopsies in the lower pole of the left kidney were performed by the same experienced team composed of nephrologist performing hands-free PRB with a radiologist showing real-time USG image and tracking of the needle. Automated devices (BARD MAGNUM Reusable Core Biopsy System, C.R BARD Inc.) with a 14-gauge and 16 cm long needle with a penetration depth of 22 mm were employed for renal biopsy.

Prior to the biopsy, an assigned channel for needle trajectory was anesthetized locally from the skin surface to the kidney capsule with a 2 % lignocaine solution. During the PRB, only one biopsy needle pass was performed.

Immediately after the biopsy, a compressive bandage was applied on the left lumbar area; then, the patient remained in the supine position for the next 24 h, with a roll on the left lumbar region for the first 4 h after PRB.

Ultrasound examinations of the left kidney (Logiq P6, Convex 3.5–5 MHz) were performed by only one independent and always the same ultrasound specialist after approximately 24 h after PRB. Left kidney and the perirenal area were visualized in 2D using posterolateral or the left lumbar area access, in the longitudinal and transverse plane. Color Doppler and power Doppler options were employed for the flow diagnosis in the observed hematomas.

**Fig. 1:**
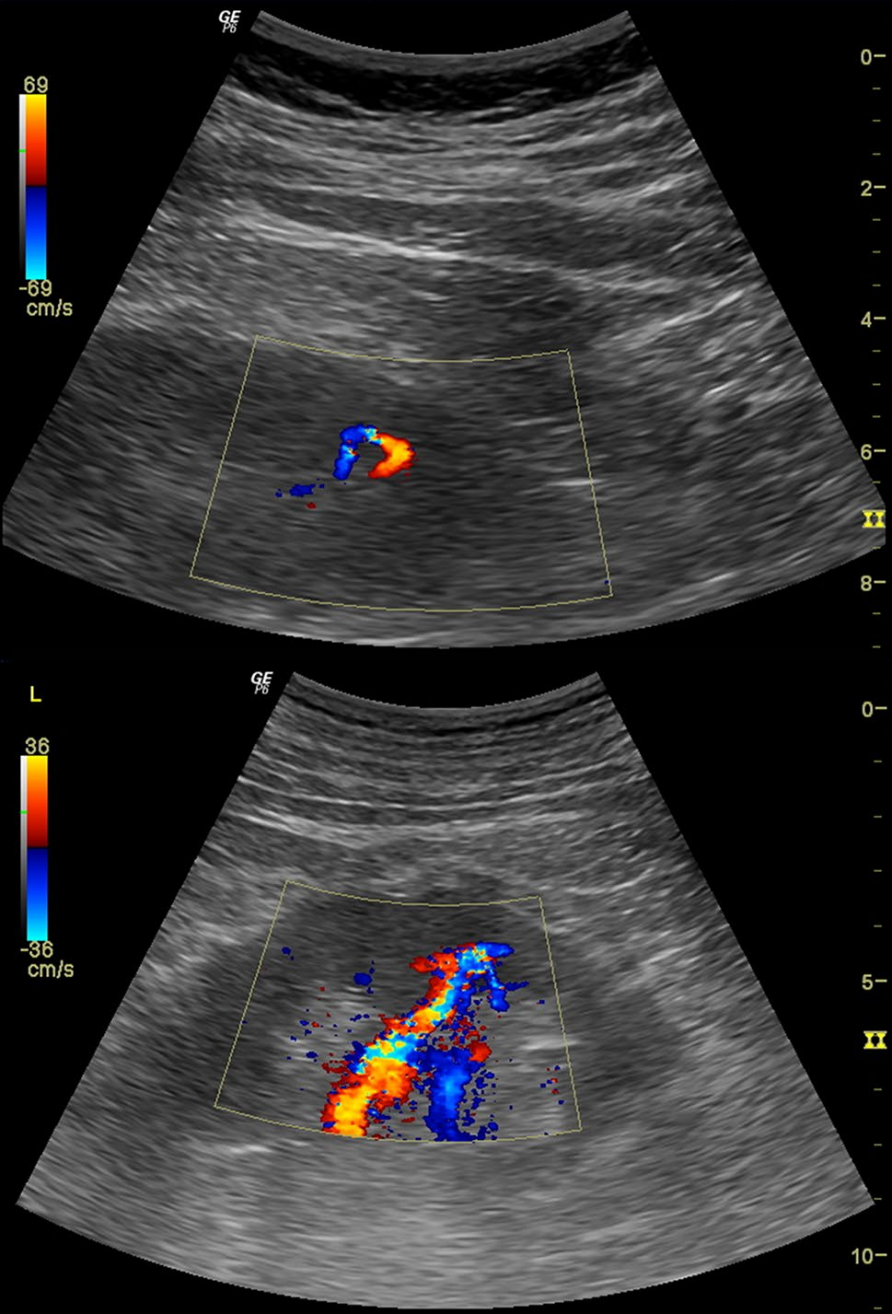
Renal biopsy-related arteriovenous fistulas detected in color Doppler

**Fig. 2:**
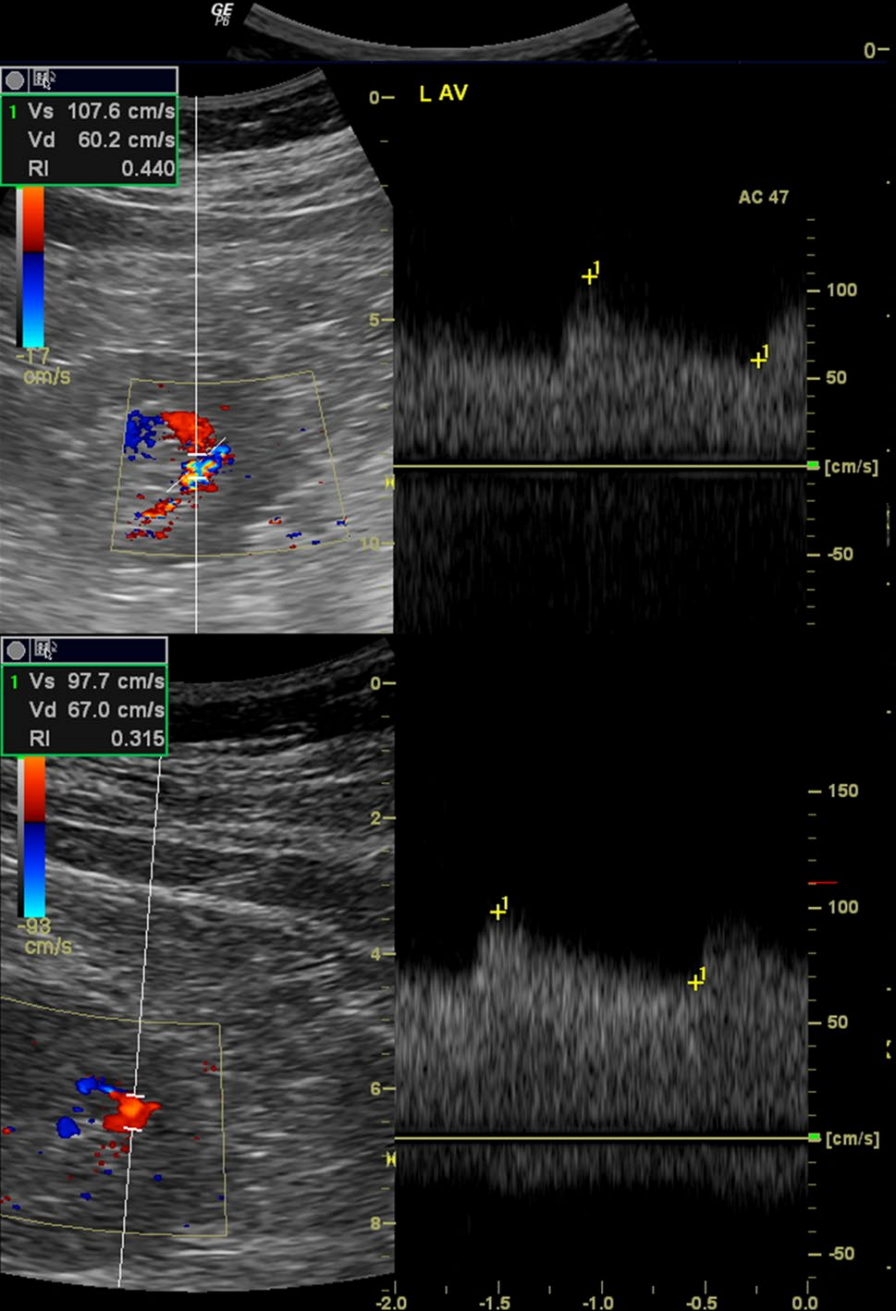
Doppler hemodynamic assessment of renal biopsy-related arteriovenous fistulas

For the diagnosis of PRB-related iatrogenic arteriovenous fistula, in CD imaging intrarenal arteries were visualized in the renal sinus and parenchyma in longitudinal sections. Then, CD velocity was gradually increased until no flow signal in the most of previously visualized intrarenal arteries was observed. Identification of high-intensity flow in one or one of several other vessels raised a suspicion of arteriovenous fistula (Fig. 1). In order to confirm the RB-AVF, maximal flow velocity (FV) and resistance index (RI) were measured in the segmental artery feeding the fistula and in the segmental artery of the middle part or of the upper pole of the left kidney, depending on the possibilities to visualize vessels (Fig. 2).

In the case of ultrasonographic characteristics of RB-AVF, patients were monitored during consecutive days of the same hospitalization, and in the course of subsequent hospitalizations if possible.

Collected in renal biopsies, tissue specimens were histologically evaluated. The number of the glomeruli in each biopsy was counted. For quantitative evaluation of interstitial inflammatory infiltration, interstitial fibrosis, tubular atrophy, arterial narrowing and hyalinosis, the recommendations of the Banff classification were used [6, 7].

## Statistical analysis

The examined variables were analyzed with Pearson’s or Spearman’s correlation test as determined by meeting the condition of normal distribution. The *t* test and *U* Mann–Whitney test were used to analyze the difference between the groups. Receiver operating characteristic (ROC) analysis was performed to assess a prognostic value of glomeruli number and maximal hematoma diameter in recognizing renal biopsy-related arteriovenous fistula. For statistical analysis, Statistica 12 (StatSoft Inc.) software was used.

## Results

Among 138 PRB, bleeding complications in the form of perirenal and subcapsular hematomas were found in 136 patients (98.55 %). The mean of maximal hematoma diameter was 36.7 ± 21.3 mm.

We observed 7 cases (0.051 %) of additional hematomas in the perirenal area or in the left iliopsoas muscle, 2 cases (0.015 %) of enlarging hematomas in subsequent examinations (including one with RB-AVF), without signs of an active bleeding, and 1 (0.007 %) active retroperitoneal hemorrhage with forming a pseudoaneurysm, which required an endovascular invasive treatment.

In the analyzed material of 138 biopsies, a total of 23 RB-AVF cases (16.67 %) were found. Groups with and without RB-AVF did not differ significantly in terms of studied demographic, biochemical and morphological data (Table 1). The maximal hematoma diameter was significantly longer in the RB-AVF group (*p* = 0.005).

**Table 1:** Comparison of demographic, biochemical and 2D ultrasound data between groups with and without renal biopsy-related arteriovenous fistula

	RB-AVF (*n* = 23)	PRB without fistula (*n* = 115)	*p* value
Age (years)	40.5 ± 14.7	47.2 ± 15.1	0.058
BMI (kg/m^2^)	25.3 ± 4.5	26.7 ± 4.5	0.165
Creatinine (mg/dl)	1.60 ± 1.51	1.67 ± 1.18	0.319
Proteinuria (g/24 h)^a^	1.60 [0.13–13.4]	3.16 [0.00–29.50]	0.073
Renal length (mm)	117.5 ± 5.2	116.0 ± 13.1	0.696
Parenchymal thickness	16.3 ± 2.9	16.0 ± 3.2	0.750
Maximal hematoma diameter (mm)	48.7 ± 23.9	34.5 ± 19.9	0.005

^a^ Median [range]

Compared to the PRB without a fistula, RB-AVF group had a significantly lower number of renal glomeruli in biopsy samples (*p* = 0.015) (Table 2). Other results of renal biopsy did not differ significantly between the groups.

**Table 2:** Comparison of renal biopsy findings between groups with and without renal biopsy-related arteriovenous fistula

	RB-AVF	PRB without fistula	*p* value
Number of glomeruli	11.9 ± 7.1	16.9 ± 9.6	0.015
Interstitial inflammation^a^	1 [0–4]	2 [0–4]	0.511
Interstitial fibrosis^a^	2 [0–4]	2 [0–4]	0.599
Tubular atrophy^a^	2 [0–4]	2 [0–4]	0.725
Arterial narrowing^a^	1 [0–3]	1 [0–3]	0.714
Arterial hyalinosis^a^	0 [0–4]	0 [0–4]	0.469

*RB-AVF* renal biopsy-related arteriovenous fistula, *RB* renal biopsy

^a^Median [range]

In the evaluated material, Doppler study showed that flow velocities were significantly higher, and RI significantly lower in arteries supplying the fistula compared to the other segmental arteries of the left kidney (Table 3). The mean difference in FV was 45.9 cm/sec and in RI 0.252. In the ROC analysis, RI of 0.524 allowed for recognizing RB-AVF with a sensitivity of 91 % and a specificity of 100 % (AUC 0.998, *p* < 0.001) and was a better predictor of this complication (*p* < 0.001) than maximum flow velocity (sensitivity 80 %; specificity 89.5 % for FV 60.9 cm/s; AUC 0.900, *p* < 0.001).

**Table 3:** Results of Doppler study on renal biopsy-related arteriovenous fistulas

	Normal renal segmental artery	Renal segmental artery supplying RB-AVF	*p* value
RI	0.673 ± 0.052	0.439 ± 0.069	<0.001
FV (cm/s)	43.43 ± 12.15	86.25 ± 31.92	<0.001
dRI^a^	0.252 ± 0.104 [range 0.132–0.615]		–
dFV (cm/s)^a^	45.9 ± 20.0 [range 4.0–97.9]		–

*RB*-*AVF* renal biopsy-related arteriovenous fistula, *RI* resistive index, *dRI* the difference between resistive indexes in normal and feeding arteriovenous fistula segmental arteries, *FV* maximal flow velocity, *dFV* the difference between maximal flow velocities in feeding arteriovenous fistula and normal segmental arteries

^a^Median [range]

**Fig. 3:**
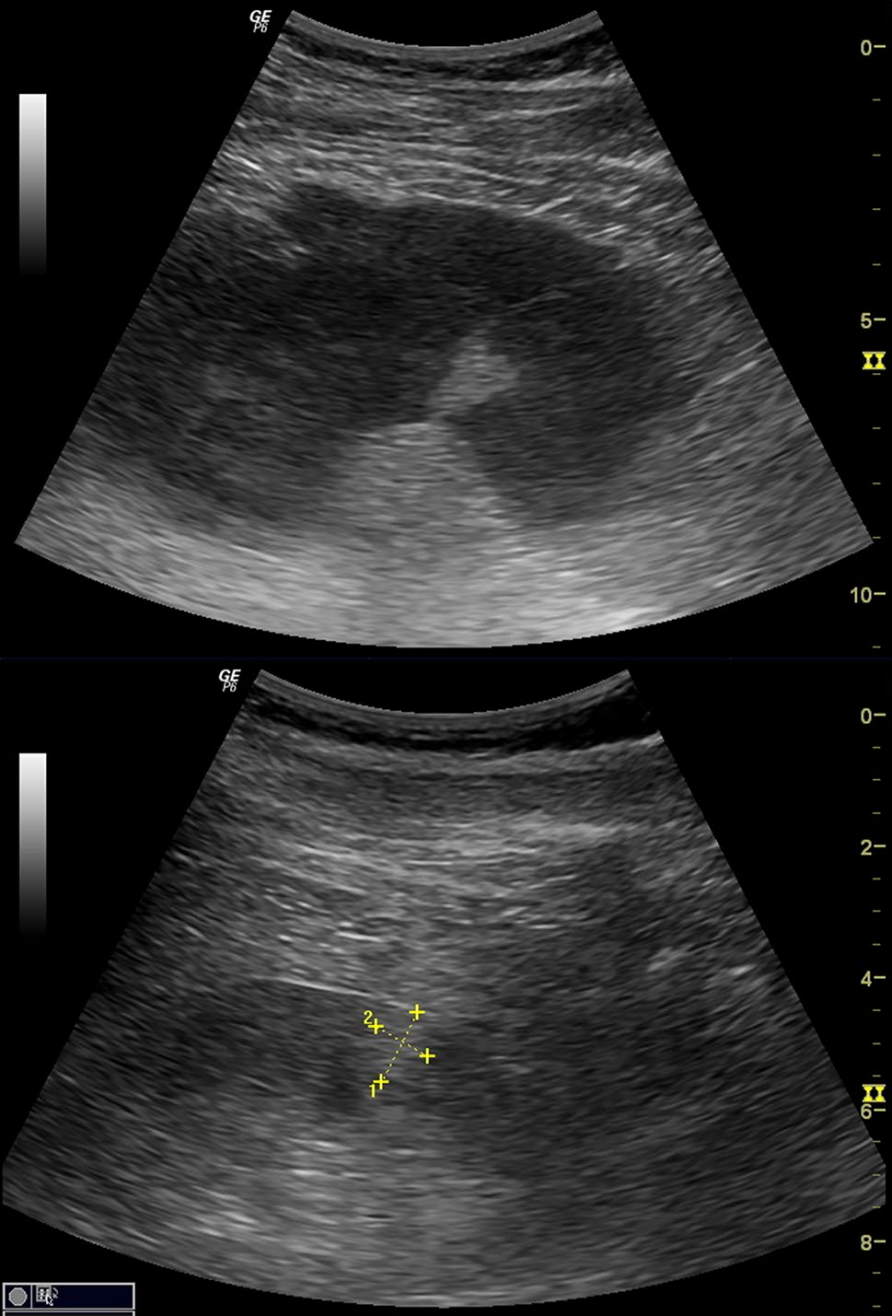
Hyperechogenic area in the neighborhood of the arteriovenous fistula

In approximately 39 % of cases (9/23), ultrasound found a conical hyperechogenic area with a mean size of 12.2 × 17.6 mm in the vicinity of the observed arteriovenous fistula (Fig. 3).

In the correlation analysis, regarding demographic, biochemical, ultrasound and histopathological results, only the number of glomeruli (*r* = −0.22) and the maximal hematoma diameter after PRB (*r* = 0.24) were significantly associated with RB-AVF (*p* < 0.05). Age, weight, BMI, kidney size, renal parenchymal thickness and creatinine were not associated with the development of the RB-AVF. In the ROC analysis, a number of glomeruli equal to 10 was associated with the occurrence of RB-AVF with a sensitivity of 60 % and a specificity of 67 % (AUC 0.673, *p* = 0.009), while the maximal hematoma diameter of 34 mm allowed for the identification of RB-AVF with a sensitivity of 65 % and a specificity of 60 % (AUC 0.686, *p* = 0.002).

In 12 cases (52 %), the persistence of arteriovenous fistula for more than 1 day was observed. The median of the period in which the presence of the fistula was found in these patients was 2 days (range 2–26 days after PRB). In 9 patients (39 %), of whom only four patients had RB-AVF for more than 1 day, the next study showed no presence of the fistula, and the median of the period from PRB to the examination was 3 days (range 2–42 days). Five patients with diagnosed asymptomatic RB-AVF were discharged from the hospital and did not report for the further study.

## Discussion

In the available literature, the main factors increasing the risk of serious complications after PRB include, inter alia, elevated creatinine concentration, systolic and diastolic blood pressure values, decreased hemoglobin concentration, coagulation disorders, the thickness of the biopsy needle (16 or 18 gauge instead of 14 gauge) and the lack of use of ultrasound-guided needle [1, 8]. Based on previous reports, it is believed that the number of serious bleeding complications requiring transfusion is 0.3–7.4 % and minor complications (small hematomas, arteriovenous fistulas, transient hematuria) 1–92 % [9, 10]. Significant discrepancies in the described complications reported by various authors probably result from the different nature of the studies and different diagnostic criteria [1]. For instance, Waldo et al. [11] reported only clinically symptomatic hematomas, but of the maximal diameter under 1 cm, while Hergesell et al. [12] considered small hematomas only from 2 × 2 cm upwards. On the basis of renal biopsy performed in 50 children, Riccabona et al. [9] found the occurrence of post-biopsy hematomas with the incidence of 92 % and arteriovenous fistulas with 12 %. Based on our prospective evaluation, we proved that PRB bleeding complications are present in a majority of procedures, but are clinically insignificant. In the ultrasound of 138 kidneys after the PRB using a 14-gauge biopsy needle, we found the presence of hematomas in up to 98.5 %. Although in our study most patients had hemorrhagic complications in the form of subscapular or perirenal hematoma after PRB, only 9 cases (6.5 %) required prolonged hospitalization and further noninvasive diagnostic procedures. In addition, only one patient (0.007 %) with an active retroperitoneal hemorrhage with forming a pseudoaneurysm required an endovascular invasive treatment. Moreover, in only one patient the occurrence of arteriovenous fistula was in relation with hematoma enlargement in subsequent examinations. These data confirm that the procedure of percutaneous renal biopsy is quite safe. On the other hand, there are data suggesting a greater safety of ultrasound-guided PRB than real-time ultrasound-assisted (hands-free) PRB [2, 8].

The presence of an arteriovenous fistula which complicates PRB both can be clinically insignificant and, in extreme cases, can cause serious hemorrhage, resistant hypertension, renal or heart failure [5, 10, 13]. The incidence of fistulas after PRB of native kidneys is estimated to be approximately 7.4–9 % and 8.3–16.6 % for transplanted kidneys, with approximately 1 of 5 RB-AVF is symptomatic [10, 12, 14, 15]. However, it should be mentioned that the transplanted kidney is easily accessible by ultrasound, and PRBs are often performed repeatedly in the same kidney. In our work, we proved a higher incidence of RB-AVF in native kidneys in comparison with the reports of other authors. The reason for this is probably the prospective nature of our study and the applied protocol assuming an active search for arteriovenous fistula after PRB. The incidence of RB-AVF in our study is comparable with the results reported in transplanted kidneys. Nevertheless, in the presented study, we could not identify any risk factors for the development of RB-AVF because it was not associated with age, weight, BMI, kidney size, renal parenchymal thickness or even with the creatinine concentration.

Although in the available literature, there are case reports on hemodynamic properties of RB-AVF, our work shows this characteristics on a significantly larger group. Based on the performed observations, we found an average twofold increase in the maximum flow velocity and RI decrease of approximately 35 % in the segmental arteries supplying the fistula. RI value ≤0.524 in the segmental artery was the best predictor of RB-AVF (sensitivity 91 %, specificity 100 %). Although Yokoyama et al. [16] observed the usefulness of ultrasound in detecting RB-AVF, they suggested it should be confirmed in CT. Schwarz et al. [14] examining RB-AVF in transplanted kidneys suggested recognition of hemodynamic relevance of this complication when the difference between RI in the renal artery and RI in the segmental artery not associated with RB-AVF does not exceed 0.05. In our observation, in all arteries supplying the fistula, RI was at least 0.1 lower compared to normal segmental arteries, which, together with the described by us technique for detection in color Doppler and qualitative assessment of turbulent flow, can be considered as a useful criterion for recognizing RB-AVF. Our results significantly expand the reports of other authors who, in ultrasound examination in patients with RB-AVF, found both an increase in the flow velocity and a decrease in RI in the artery supplying fistula, but no diagnostic thresholds have been proposed [9, 10, 15, 17]. Most similar results of hemodynamic changes in RB-AVF were demonstrated by Middleton et al. [18] on the example of eight patients, but due to the size of the group and the characteristics of the equipment at that time, the data may be partly misleading.

In the presented study, ultrasound examinations were performed by only one and always the same experienced ultrasound specialist. Moreover, a single measurement of hemodynamic parameters was performed in arteries supplying AVFs as well as in normal segmental arteries. Thus, the assessment of interobserver and intraobserver variability was not possible. Based on previous studies, in RI measurements taken by well-trained staff, intraobserver variability ranged from 2.07 to 5.1 %, while interobserver variability ranged from 3.61 to 6.2 % [19].

Performing 2D ultrasound in patients with RB-AVF, in approximately 40 % of cases we found the presence of the conical hyperechogenic area in the vicinity of the fistula, probably resulting from stealing blood by the fistula and ischemia in this fragment of parenchyma. To the best of our knowledge, this ultrasound phenomenon has not been thus far described in the available literature.

Demonstrated in our study, the correlation of fewer glomeruli in PRB and, at the same time, larger sizes of bleeding complications in patients with RB-AVF suggests probably insufficient control of the biopsy site in a hands-free procedure, which may result from both the change in the direction of the needle at the time of releasing a biopsy mechanism. The use of ultrasound-guided biopsy needle could probably reduce the incidence of bleeding complications and increase the diagnostic value of PRB [8]. The other, but direct reason for the diminished number of glomeruli in RB-AVF patients could be perivascular and vascular area accessed by the biopsy needle.

In the available reports, most RB-AVFs close spontaneously. Brandenburg et al. [15], examining RB-AVF in transplanted kidneys, observed a spontaneous closure of 50 % fistulas within 2 days, and 75 % of RB-AVFs during 4 weeks. Nevertheless, the authors reported a persistence of 3 fistulas (25 %) for more than 1 year. Our results seem to coincide with this report, because although we observed a spontaneous closure of RB-AVF after an average of 3 days in approximately 40 % of patients, five patients (21.7 %) with RB-AVF were not subjected to further observation for objective reasons. The above-mentioned lack of the data does not allow for adequate conclusions and is a limitation of our work. Due to the fact that most of RB-AVFs are asymptomatic and resolve spontaneously, in these cases a follow-up Doppler sonogram, about 6 months after renal biopsy, could help in the detection of persisting and progressing arteriovenous fistulas [20].

## Conclusions

Bleeding complications are observed in most of real-time ultrasound-guided native renal biopsies, but in a majority of cases, their clinical significance is low. Post-biopsy arteriovenous fistulas occur with a similar incidence in native and transplanted kidneys. They can cause a local steal syndrome corresponding to the fragment of parenchyma, detectable in the 2D ultrasound. The arteries supplying the fistula are characterized by an increased flow velocity and a reduced resistance index, which can be a useful tool in the detection of arteriovenous fistulas. Although percutaneous renal biopsy still remains a safe procedure and most of post-biopsy arteriovenous fistulas are clinically silent, a follow-up Doppler sonography in a few months after renal biopsy is proposed to discover persisting and progressing arteriovenous fistulas.
